# Genetic analysis reveals unique characteristics of *Plasmodium falciparum* parasite populations in Haiti

**DOI:** 10.1186/s12936-020-03439-7

**Published:** 2020-10-23

**Authors:** Rachel F. Daniels, Stella Chenet, Eric Rogier, Naomi Lucchi, Camelia Herman, Baby Pierre, Jean Frantz Lemoine, Jacques Boncy, Dyann F. Wirth, Michelle A. Chang, Venkatachalam Udhayakumar, Sarah K. Volkman

**Affiliations:** 1 Department of Immunology and Infectious Diseases, Harvard T.H. Chan School of Public Health, Harvard T.H. Chan School of Public Health, 665 Huntington Ave, Boston, MA 02115 USA; 2 Broad Institute, Cambridge, MA USA; 3 Malaria Branch, Division of Parasitic Diseases and Malaria, Centers for Disease Control and Prevention, Atlanta, GA USA; 4 CDC Foundation, Atlanta, GA USA; 5 Ministère de La Santé Publique Et de La Population (MSPP), Programme National de Contrôle de La Malaria, Port-au-Prince, Haiti; 6 Instituto de Medicina Tropical, Universidad Nacional Toribio Rodríguez de Mendoza de Amazonas, Chachapoyas, Peru

**Keywords:** *Plasmodium falciparum*, Malaria elimination, Haiti, Hispaniola, Genetic analysis, Population genetics

## Abstract

**Background:**

With increasing interest in eliminating malaria from the Caribbean region, Haiti is one of the two countries on the island of Hispaniola with continued malaria transmission. While the Haitian population remains at risk for malaria, there are a limited number of cases annually, making conventional epidemiological measures such as case incidence and prevalence of potentially limited value for fine-scale resolution of transmission patterns and trends. In this context, genetic signatures may be useful for the identification and characterization of the *Plasmodium falciparum* parasite population in order to identify foci of transmission, detect outbreaks, and track parasite movement to potentially inform malaria control and elimination strategies.

**Methods:**

This study evaluated the genetic signals based on analysis of 21 single-nucleotide polymorphisms (SNPs) from 462 monogenomic (single-genome) *P. falciparum* DNA samples extracted from dried blood spots collected from malaria-positive patients reporting to health facilities in three southwestern Haitian departments (Nippes, Grand’Anse, and Sud) in 2016.

**Results:**

Assessment of the parasite genetic relatedness revealed evidence of clonal expansion within Nippes and the exchange of parasite lineages between Nippes, Sud, and Grand'Anse. Furthermore, 437 of the 462 samples shared high levels of genetic similarity–at least 20 of 21 SNPS–with at least one other sample in the dataset.

**Conclusions:**

These results revealed patterns of relatedness suggestive of the repeated recombination of a limited number of founding parasite types without significant outcrossing. These genetic signals offer clues to the underlying relatedness of parasite populations and may be useful for the identification of the foci of transmission and tracking of parasite movement in Haiti for malaria elimination.

## Background

The elimination of malaria is a current goal of the countries comprising the island of Hispaniola; namely, Haiti and the Dominican Republic. This effort is part of an overarching goal by these countries to create a malaria-free zone across the Caribbean by 2025 [1]. The main malaria-causative parasite in this region is chloroquine-susceptible *Plasmodium falciparum* transmitted mainly in Haiti by *Anopheles albimanus* mosquitoes [2]. Overall, malaria transmission in Haiti is low, with approximately 21,000 confirmed cases in 2016, the year when this study’s samples were collected, and a nationwide prevalence of 0.4% [3, 4]]. The largest number of cases is reported in the southwestern Departments, particularly near coastal areas [5]. As part of ongoing malaria elimination efforts, surveys have been carried out to understand the overall prevalence and transmission foci of the disease in Haiti.

In addition to the standard epidemiological measures of program progress, genetic tools offer additional understanding of *Plasmodium* populations to help in tracking parasite movement and determining the impact of interventions in shrinking the *Plasmodium* reservoir. The level of genetic diversity and its distribution could provide insights into parasite transmission and parasite population history. Moreover, genetic variation can be used to uniquely identify parasites that infect individuals in a particular region, establishing clusters of parasite lineages that can be followed over time and space after interventions are applied [6–8].

In this context, the present study assessed the genetic diversity of samples collected during 2016 as a baseline for understanding current population structure in the selected study sites and providing additional information for the future development of a molecular database in Haiti that also includes drug resistance markers. The availability of such a molecular database, with the hope of adding future data collections, may be useful for various programme-related uses, such as tracking parasite movement within the country (e.g., parasite sink and source), evaluation of the efficacy of intervention strategies, and outbreak investigations which help the national programme to apply programme-level applications to progress towards the goal of malaria elimination.

## Methods

### Study sites

Haiti, with an estimated population of 10,788,440 in 2016, occupies the western one-third of the island of Hispaniola located between the Caribbean Sea and the North Atlantic Ocean and shares a border with only the Dominican Republic [3, 9]. The country is divided into 10 Departments subdivided into approximately 144 communes and 571 communal sections.

Malaria is transmitted primarily through the rainy seasons in Haiti from May through July and again from October through November. Rainfall is variable, ranging from 400 to more than 3000 mm annually, with the reported incidence of malaria heterogeneously distributed in foci or “hot-spots” primarily in the coastal areas of the Departments located in the southeastern part of the country [10].

### Sample sites and collection

The sample sites included four sentinel sites in three departments (Grand’Anse, Sud, and Nippes) from among 11 sites nationwide established for anti-malarial molecular resistance marker surveillance, as described elsewhere [11]. Briefly, individuals of all ages seeking treatment at clinics located at the sentinel sites with symptoms of malaria who also tested positive for malaria by either microscopy or rapid diagnostic test (RDT) from March 2016 to December 2017 were considered eligible to participate. Microscopy was performed by experienced microscopists from thick-smear Giemsa-stained slides. RDT testing was performed with the HRP2-based commercial kits available at each health facility: First Response Malaria Ag HRP2 (Premier Medical Corporation Ltd., Watchung, New Jersey, USA), CareStart Malaria HRP2 Pf (Access Bio, Inc., Monmouth Junction, New Jersey, USA), or SD Bioline Malaria Ag Pf (Standard Diagnostics, Inc., Yongin, Korea). Interviews were also conducted to collect patient information (including age, gender, recent travel history). Patients who were clinically unstable or requiring urgent medical care were excluded from participation. The protocol for molecular surveillance was approved by the Haitian Ministry of Public Health and Population Bioethics Committee as a non-research programmatic activity. This protocol was also reviewed by Center for Global Health of U.S. Centers for Disease Control and Prevention (CDC) and approved as a non-research surveillance activity. Blood specimens were collected only when participants or the parents or guardians of children provided verbal consent to participate.

Approximately 200 μL of blood were obtained by fingerprick from each consenting participant, placed on Whatman 903 Protein Saver cards (GE Healthcare), and allowed to dry overnight before storage in an individual plastic bag with desiccant. To ensure consistency in blood sample collection, all health facility workers had previously been trained on appropriate dried blood spot (DBS) sample preparation. Filter papers were stored at health facilities at room temperature away from sunlight.

### Sample extraction and molecular genotyping

Genomic DNA was isolated from dried blood spots using QIAamp DNA blood mini kits (Qiagen, Valencia, CA, USA) according to manufacturer’s protocol except that double the amount of filter paper blood and proteinase K were used. After elution, DNA samples were kept at 4 °C and −40 °C for short- and long-term storage, respectively.

The samples were pre-amplified and genotyped for 24 genome-wide, putatively neutral single-nucleotide polymorphisms (SNPs) using a TaqMan-based barcoding protocol as previously described [12] on a VIIA real-time PCR system (Thermo Fisher Scientific, Foster City, CA, USA). Briefly, the extracted patient samples containing parasite genetic material underwent pre-amplification in a reaction mixture containing 0.2 × of the primer concentrations used for standard *P. falciparum* molecular barcode genotyping in the 2 × TaqMan PreAmp Master Mix (Applied Biosystems cat# 4391128). The resulting products were cleaned (ZR-96 DNA Clean-Up Kit, Zymo Research cat# D4017) and used as template in 5-uL TaqMan molecular barcode assays. The genotypes were determined from the raw data reads downloaded from the instrument using an in-house script (available and documented at https://github.com/ndaniels/TaqmanParser).

### Data and statistical analysis

**Fig. 1:**
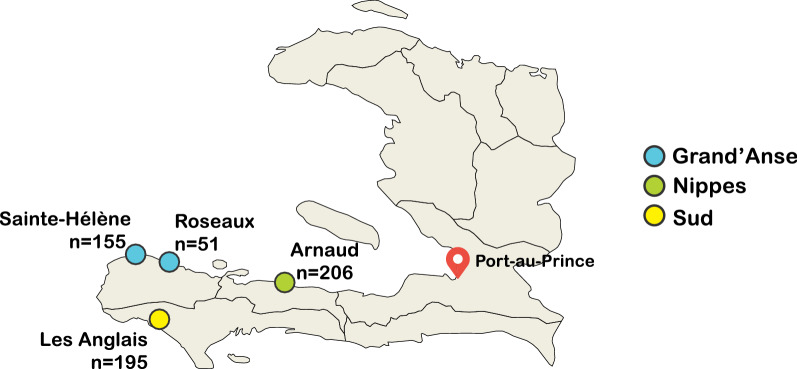
Sites and distributions of Haitian samples included in the present study. The sample sites included four sentinel sites in three departments (Grand’Anse, Sud, and Nippes) from among 11 sites nationwide established for anti-malarial molecular resistance marker surveillance. A total of 648 samples were collected from consenting microscopy or RDT-positive individuals at the clinic sites in Grand’Anse, Sud, and Nippes and submitted for genetic analysis

A total of 648 samples were collected and submitted for genetic analysis. All samples were collected from consenting microscopy or RDT-positive individuals at the clinic sites in Grand’Anse, Sud, and Nippes (Fig. 1). The samples were analysed using the genotypic information of complete SNPs barcodes. Samples with > 5 missing calls in the 24-SNP molecular barcode were removed from analysis as failed barcodes. A total of 21 informative SNPs was included in the analysis.

Monogenomic 21-SNP barcodes were included in the pairwise comparisons in analysis of identity-by-state (IBS) to identify samples that were identical and highly-related [13]. In this study, ‘identical’ was defined as IBS at all 21 SNP positions, while ‘highly-related’ was defined as IBS at 20 of the 21 SNP positions based on previous work comparing molecular barcodes to whole-genome sequencing data [8].

Additionally, spatial principal components analysis (sPCA) was performed for comparison to other populations in Central and South America using the adegenet function within the PopGenReport package version 2.0 in R version 3.02 (R Foundation for Statistical Computing, Vienna, Austria) [14].

The number of base differences per sequence from the mean diversity for each site were calculated [15]. Standard error estimate(s) were obtained by a bootstrap procedure (500 replicates) in MEGA7 [16]. The significance of differences between populations was assessed by Fisher’s exact tests. For all statistical analyses, p < 0.01 was considered statistically significant.

## Results

### Patient characteristics

The patient demographics (age, gender, department/health facility, and travel history) are shown in Additional file 1: Table S1. Of the samples eligible for analysis, as described below, 195 of the patients were men and 266 were women (one patient was missing data on gender). The gender distribution was particularly skewed for Nippes, with 118 women and 51 men. This skew may have been due to the fact that Nippes is the only health institution in this area and provides pre-natal, maternal child health services, immunizations, and general medical services. Thus, this clinic may attract a higher proportion of women since they have fewer options. The institutions in other areas provide similar services, but the population they serve have access to other facilities. Overall, the patient ages ranged from less than 1 year to 86 years of age, with the highest proportion of participants in the 16–20-years age group for all departments included in the present study.

### Samples included in the analysis

**Fig. 2:**
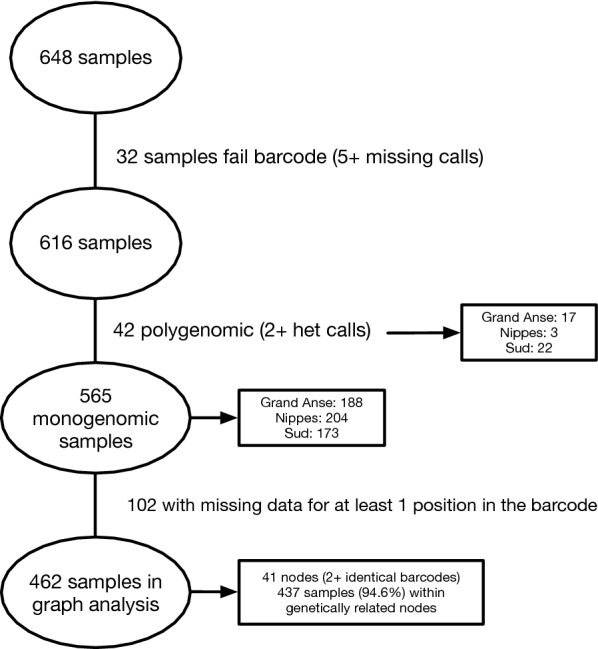
Sample flowchart showing the inclusion of samples in the current analysis. Of the 648 *P. falciparum* samples collected during 2016 in Haiti for this study, 32 (4.9%) were excluded for 5 or more missing SNP calls. Of the remaining 616 samples, 42 (6.8%) were polygenomic (multiple distinct genomes, defined as the presence of both bi-allelic alleles at two or more positions in the SNP barcode). From the remaining 565 monogenomic (single distinct genome) samples, 462 with complete data for the 21 most informative SNPs were included in the subsequent analyses

Of the 648 *P. falciparum* samples collected during 2016 in Haiti for this study, 32 (4.9%) were excluded for 5 or more missing SNP calls. Of the remaining 616 samples, 42 (6.8%) were polygenomic (multiple distinct genomes, defined as the presence of both bi-allelic alleles at two or more positions in the SNP barcode). From the remaining 565 monogenomic (single distinct genome) samples, 462 with complete data for the 21 most informative SNPs were included in the graph analysis (Fig. 2). The samples were collected in three Departments: Grand’Anse (34.2%), Sud (31.6%), and Nippes (32.5%).

### sPCA

**Fig. 3:**
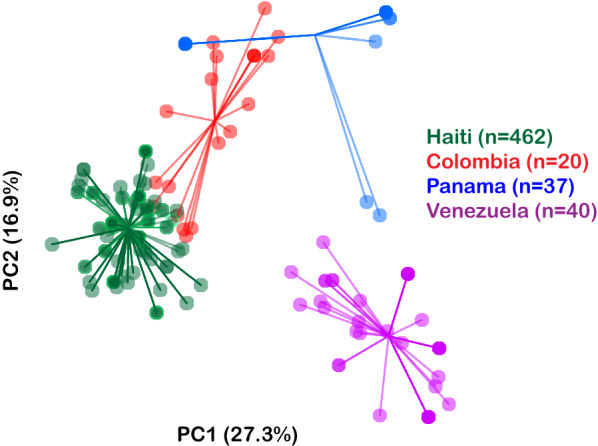
Spatial principal components analysis (PCA) comparing the genetic relatedness of these parasites in the southern area of Haiti and their relationship to other parasite populations from surrounding countries. The plot includes the 21-SNP molecular barcodes of *P. falciparum* infections from neighboring countries of Central and South America and shows differentiation between the parasite population from Haiti and those of the other countries

To compare the genetic relatedness of these parasites in the southern area of Haiti and their relationship to other parasite populations, spatial principal components analysis was performed of the 21-SNP molecular barcodes of *P. falciparum* infections from neighbouring countries of Central and South America in a [17], which showed differentiation between the parasite population from Haiti and those of the other countries (Fig. 3). These findings suggested that an ancestral Haitian *P. falciparum* population is responsible for current transmission of malaria within the country.

### Genetic relatedness

**Fig. 4:**
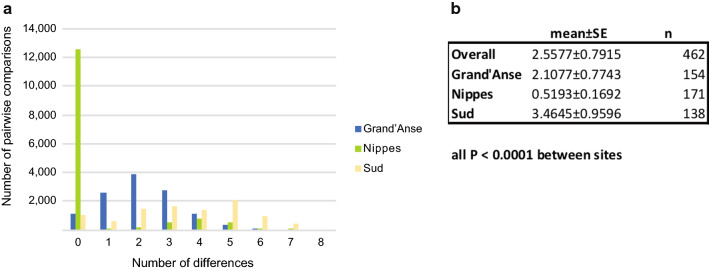
Analysis of the distributions of the numbers of pairwise differences in 21-SNP barcodes between Departments (A) and the statistical significances of the differences between mean overall numbers of differences and for each Department (all p < 0.0001, Fisher’s exact tests)(B)

The overall genetic diversity (pi) was 4.881 standard error (SE) 0.824. Comparisons of the genetic diversity between departments revealed similar levels between Grand’Anse and Sud (4.568 SE 0.887 and 5.504 SE 0.897, respectively), with significantly lower genetic diversity in Nippes (0.964 SE 0.208**)** (p < 0.001). In contrast, analysis of the distributions of the number of pairwise differences in 21-SNP barcodes between sites revealed significant differences for all pairwise comparisons, although the magnitude of the difference was greatest between Nippes and the other sites (all p < 0.0001) (Fig. 4).

### Graph analysis

**Fig. 5:**
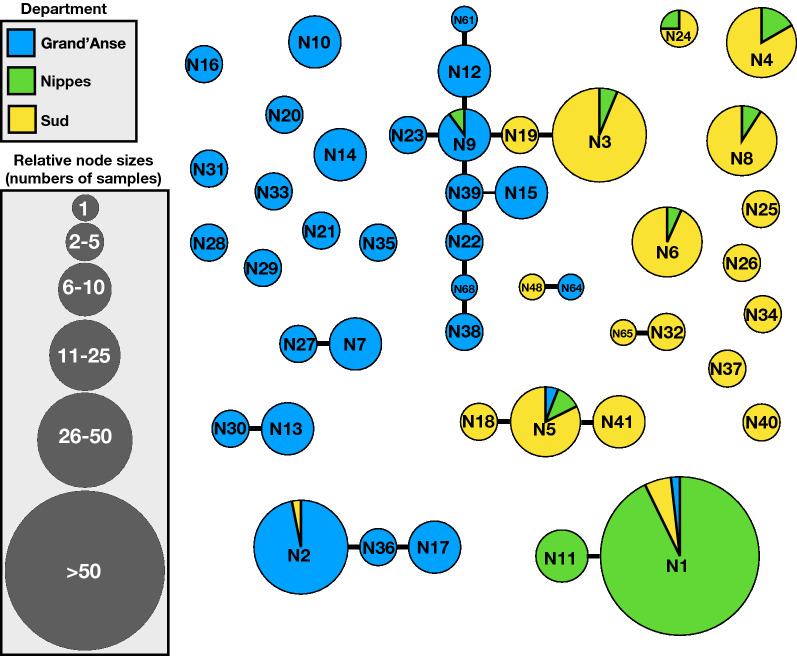
Graph analysis of all 462 samples with complete 21-SNP barcodes. The barcode-identical samples are grouped into nodes (circles) connected by edges (lines) only to nodes with which they share 20 of the 21 barcode sites. Among the 41 barcode-identical nodes (sized relative to the number of samples sharing the same barcode), 28 were highly related to another barcode-identical node (sharing 20 of the 21 barcode SNPs). Only 25 samples (6%) are not included among the identical and highly-related nodes. The ‘N’ numbers correspond to the node numbers indicated in Additional file 1: Table S1

IBS analysis revealed that 437 of 462 monogenomic samples (94.6%) were identical or highly related to at least one other sample (p < 0.0002 for the likelihood of 0 or 1 differences by chance, contingency table). The relationships between these samples were then graphed. As shown in Fig. 5, barcode-identical samples are grouped into nodes (circles) connected by edges (lines) only to nodes with which they share 20 of the 21 barcode sites. Among the 41 barcode-identical nodes (sized relative to the number of samples sharing the same barcode), 28 were highly related to another barcode-identical node (sharing 20 of the 21 barcode SNPs) (Fig. 5). Only 25 samples (6%) were not included among the identical and highly-related nodes. The ‘N’ numbers correspond to the node numbers indicated in Additional file 1: Table S1.

The distribution of the number of nodes in each site was as follows: Grand’Anse 26, Nippes 9, and Sud 17. Grand’Anse had two collection sites, Sainte-Hélène (n = 155) and Roseaux (n = 51) with 25 and 10 barcode-identical nodes, respectively. Although the number of samples from each site differed, the same node (N2) was dominant in both sites. This node was unique to Grand’Anse except for one sample found in Sud, which also had the same barcode profile. Additionally, nodes were shared between Grand’Anse and Sud (N2), Grand’Anse and Nippes (N9), Nippes and Sud (N3, 4, 6, 8, and 24), and all three sites (N1 and N5). Only 9 nodes were observed in Nippes and 95% of the samples from this department belonged to the same node (N1), which was also included samples from Grand’Anse and Sud (Additional file 1: Table S1).

Examination of the graph properties revealed one connected component with a maximum graph diameter of 6 (N38, 68, 22, 9, 12, and 61 or N38, 68, 22, 39, 9, 19, and 3), with the next-highest diameters of 2–3.

## Discussion

In 2016, an estimated 21,998 cases of malaria were reported in Haiti among a population of 10.8 million [4]. The goal of malaria elimination in Hispaniola in the foreseeable future was supported by Malaria Zero, an alliance of partners including the Haitian Ministry of Health. In the preparatory operational phase of the country’s elimination plan, efforts are focused on identifying areas with ongoing relatively high transmission and risk of infection through improved surveillance, supplemental surveys as well as the development of new approaches to elimination. Once the high transmission areas are identified, targeted interventions such as targeted mass drug administration and indoor residual spraying could be deployed as additional interventions. The use of parasite genomic data could assist in prioritizing and sequencing high transmission areas for the additional interventions.

In addition to the traditional markers of transmission such as epidemiological signals, genetic analysis offers additional information about parasite drug resistance as well as the underlying parasite population structure. Genetic signals can be used for the identification and characterization of the *P. falciparum* parasite population, to identify foci of transmission, detect outbreaks, and track parasite movement [18, 19].

The present study analysed more than 600 samples collected in 2016 from three Departments in Haiti as part of an effort to characterize the parasite population in these regions. Based on its barcodes, Haiti’s parasite population was distinct from those of neighbouring countries in Central and South America, indicating that *P. falciparum* parasites are not commonly imported from neighbouring countries to Haiti and that the residual ancestral Haitian *P. falciparum* population is responsible for current transmission of malaria.

The low proportion of polygenomic infections supports the historically relatively low transmission rates reported in the country [4]; however, the presence of polygenomic infections also indicates the potential for localized regions with increased risk of multiple infectious bites or co-transmission of two parasite types [20]. The low number of polygenomic infections in this area might also give some indication of a mosquito barrier for co-transmission of multiple infections. However, the lack of reported travel history between sites further supports local transmission with limited outcrossing between parasite types (Additional file 1: Table S1).

The results also revealed similar and low levels of genetic diversity between Grand’Anse and Sud, each with a number of individual barcode-identical nodes. Nippes, however, had reduced genetic diversity compared to those of the other two sites, with an expanded node of barcode-identical parasites. Although the parasites were similar within Haiti, they were distinct from those sampled from South and Central America.

The overall low diversity in this population is suggestive of repeated, long-term inbreeding of parasite types or the expansion of a single clone or limited number of lineages due to differences in reproductive success due to host or vector-related factors, host or vector immune invasion, or other stochastic factors in this region.

While the genetic diversity (pi) was comparable between Sud and Grand’Anse, the number of pairwise differences differed significantly between Departments, with a lower mean number of differences in Grand’Anse (2.1077 ± 0.7743) than that in Sud (3.4645 ± 0.9596), suggesting differences between these parasite populations such as focal transmission (hotspots) or higher levels of crossover of parasite types, respectively.

In addition to aggregate statistical analysis, graph analysis revealed trends in the sample population consistent with long-term population inbreeding. The high proportion of related nodes within samples from Grand’Anse, Nippes, and Sud suggested that residual ancestral Haitian parasites primarily contribute to malaria transmission in this country. However, the limited number of parasites more distantly or not genetically related to these nodes may also themselves be informative. For example, they may represent evidence of local adaptation or evolution of parasites, which may become newly established lineages within the country over time.

The graph property of betweenness centrality revealed only one component [21]. This property may be more informative in comparisons of additional longitudinal sampling in the same sites, increased numbers of sampling sites nationwide, and graphs of samples from other countries and regions.

The present study used genomic data from a set of SNPs to assess both aggregate data (genetic diversity and pairwise distances) as well as individual data (clonality/unique molecular barcodes, mono/polygenomic proportions, graph characteristics) to assess the characteristics of the parasite population in this region of Haiti. Previous studies have also used different genomic markers (microsatellites) to characterize the population structure of *P. falciparum* in Haiti [5, 22]. These studies reported genetic signals including a low proportion of multiply-infected individuals (polygenomic) individuals compared to single infections (monogenomic), consistent with the findings in the present study. Carter et al*.* also observed low levels of population structure; however, they also reported high levels of genetic diversity and a lack of evidence of recent parasite population bottlenecks or increased inbreeding, in contrast to the findings of the present study. However, this difference may be due to differences in the types of markers used (microsatellites versus SNPs in the present study) [5].

While some of these discordant findings may be due to differences between studies, including study sites, collection years, patient demographics, sampling and genetic markers used, to our knowledge, few studies have reported a very large number of highly related parasites as was observed in Nippes. There are several possible explanations for this finding. First, this could indicate the emergence and spread of a specific parasite type more reproductively successful than other parasites in the population, such as the emergence of drug resistance or reduced drug sensitivity. However, a previous study monitoring drug resistance alleles in these samples [11] showed that all parasites were wild-type for *Pfcrt*, the genetic marker associated with resistance to chloroquine, the drug primarily administered in Haiti [3]. Additionally, only one mutation *Pfdhfr* S108N (associated with antifolate resistance) was found in 47% of the samples. Other unknown parasite characteristics could have contributed to the differences in relative fitness of this parasite type, including those related to human hosts and mosquito vectors. Finally, this finding may simply be a stochastic event in which a single parasite type is propagated throughout the population. Previous studies have made similar observations of clonal types in other countries in which *P. falciparum* transmission was reduced to low levels, including Peru, Ecuador, and Thailand [23–25].

The limitations of this study include potential biases due to the passive case detection and the limited number of sites, which prevent generalization of the results even within Departments without additional sampling. Moreover, not all available samples were included in the analysis.

In addition, epidemiological data, particularly longitudinal data, would be useful to place these genetic patterns in context with incidence trends and to differentiate stochastic population effects from those caused by control efforts. Furthermore, evaluation of these samples using technologies other than SNP genotyping of a limited number of markers, including whole-genome sequencing or microsatellite typing, are warranted in future studies to potentially differentiate sub-sets of samples with lower levels of relatedness or shared regions of the genome and those evolving on a shorter evolutionary timescale.

## Conclusions

The use of the population genetic approaches relies on the routine collection of parasite genetic data since the discriminatory power of the genotyping method depends on the local epidemiology and transmission setting. Since genetic signals can change over time, continued characterization of *P. falciparum* genetic diversity throughout Haiti will improve the field’s understanding of the parasite population structure and help to track movement of parasites as malaria elimination efforts progressively reduce ongoing malaria transmission in pursuit of malaria elimination in this region. These data are informative for the development of a molecular database in Haiti that includes drug resistance and other molecular markers from sites nationwide. This database, with the addition of data from future collections, may be useful for program-related activities such as tracking parasite movement within the country that could inform the prioritization and sequence of high transmission areas to be targeted, evaluation of the efficacy of intervention strategies, and outbreak investigations to understand the epidemiology and inform a response.

Population genetic data can inform these targeted control efforts, particularly when resources are limited. For instance, surveillance based on the genetic relatedness of parasites may show the emergence of a highly related or clonal population, as observed in the present study in Nippes. This genetic evidence is suggestive of local transmission, for which vector control efforts would likely be most effective. In contrast, a lack of clonality or the presence of higher proportions of polygenomic samples may suggest importation or a mobile or human population for which mass test-and-treat or other (human) drug-based measured may be warranted.

The selection of appropriate tools for genetic and genomic analysis is also based on the main goals of the national control programmes as well practical concerns such as budget. While declining costs for whole-genome sequencing appear to make this technology more attractive, as it offers ‘all the data’, the infrastructure required to support instruments and to perform analysis remain a hurdle in many settings, especially those interested in near-real-time data analysis for decision making. Furthermore, the whole-genome sequencing analysis tools for polygenomic samples require additional development to provide quantitative data on the number of genomes or the predominant alleles present in a sample. The fine-scale genetic relationships revealed by whole-genome sequencing are best suited for contexts with relatively fewer cases such that reactive case detection and follow-up can reveal more distantly related samples to track parasite movement and inbreeding across regions.

Thus, there remains a role for technologies such as the molecular barcode and microsatellite typing as more general and more easily accessible tools to identify larger-scale changes in parasite populations warranting further investigation and resources for infection control and elimination. Both SNP genotyping as used by the molecular barcode and microsatellite typing offer comparable information, although microsatellites vary on different evolutionary timescales due to their higher rates of mutation [26] which, depending on the number and identity of the microsatellites, may not be as informative as a genome-wide set of SNPs for programme-level questions regarding changes in parasite population genetics.

These programmatic considerations and uses of parasite genomic information will become more important as the number of malaria cases decline, corresponding with a decrease in genetic diversity, as the country moves towards elimination.

## Supplementary information

**Additional file 1: Table S1.** All barcodes, samples, cluster numbers for monogenomic and polygenomic sample.

## Data Availability

All data generated or analysed during this study are included in this published article and its additional file.

## Abbreviations

IBS: Identity-by-state
RDT: Rapid diagnostic test
sPCA: Spatial principal components analysis
